# Characterising C-X-C Chemokine Receptor 4 Dynamics in the Cell Membrane Using Fluorescence Fluctuation Spectroscopy

**DOI:** 10.3390/biom16081107

**Published:** 2026-07-29

**Authors:** Noemi Karsai, Joëlle Goulding, Leigh A. Stoddart, Laura E. Kilpatrick, Stephen J. Hill, Meritxell Canals, Stephen J. Briddon

**Affiliations:** 1 Division of Physiology, Pharmacology and Neuroscience, School of Life Sciences, Queen’s Medical Centre, University of Nottingham, Nottingham NG7 2UH, UK; 2 Centre of Membrane Proteins and Receptors (COMPARE), University of Birmingham and University of Nottingham, The Midlands, UK; 3 School of Pharmacy, Biomedical Discovery Institute 3, University of Nottingham, Nottingham NG7 2RD, UK

**Keywords:** C-X-C chemokine receptor 4 (CXCR4), fluorescence correlation spectroscopy (FCS), fluorescence recovery after photobleaching (FRAP), raster image correlation spectroscopy (RICS), number and brightness (N&B), photon counting histogram (PCH), G protein-coupled receptor (GPCR), fluorescence fluctuation spectroscopy (FFS)

## Abstract

The spatial organisation of plasma membrane proteins such as G protein-coupled receptors (GPCRs) plays a critical role in regulating cell signalling, function, and ultimately cell fate. Resolving this organisation requires techniques capable of probing dynamics at the single-molecule level with high spatial and temporal resolution. In this study, we employ the complementary fluorescence fluctuation spectroscopy approaches, Fluorescence Correlation Spectroscopy (FCS), Photon Counting Histogram Analysis (PCH), Raster Image Correlation Spectroscopy (RICS) and Number and Brightness Analysis (N&B), in conjunction with Fluorescence Recovery After Photobleaching (FRAP), to investigate the membrane organisation of the C-X-C chemokine receptor 4 (CXCR4), a GPCR known to undergo ligand-induced reorganisation. At the nanoscale, FCS highlighted opposing effects on diffusion after agonist (CXCL12) and inverse agonist (IT1t) treatment, whilst RICS also showed ligand-mediated changes in particle number. Both single-point and image-based brightness analyses (PCH and N&B) showed increased brightness after CXCL12 treatment, consistent with the pre-internalisation clustering of CXCR4. At the microscale, FRAP showed an increase in immobile CXCR4, not visible to FFS approaches, following CXCL12 stimulation. This integrated approach, performed on a single commercial confocal microscope, provides valuable insight into the reorganisation of CXCR4 in the plasma membrane over a range of temporal and spatial scales, which are not detectable using standard imaging.

## 1. Introduction

The plasma membrane is a dynamic structure allowing both free and directed diffusion of individual proteins and macromolecular complexes, conferring an infrastructure and organisation that has a crucial role in cell signalling and function [1,2]. G protein-coupled receptors (GPCRs) are a large and clinically relevant family of transmembrane proteins, whose organisation within the plasma membrane has been shown to influence signalling, cell function and cell fate [3,4,5]. This organisation spans several levels of complexity, from nano- to macro-domains, with intermolecular interactions lasting as short as a few microseconds [6,7,8]. Understanding this complexity requires techniques able to report dynamic events with high spatiotemporal resolution at the single cell level to dissect the single molecule responses, which can be masked within ensemble measurements.

One such set of techniques is the Fluorescence Fluctuation Spectroscopy (FFS) family, which exploit the inherent time-dependent information within the intensity signal of a detected fluorescence signal or image. This combination of approaches can be used to investigate in detail the complex membrane organisation of GPCRs such as the C-X-C chemokine receptor 4 (CXCR4). CXCR4 is a well-characterised Class A GPCR that binds its endogenous ligand, CXCL12 (also known as SDF-1α), and is involved in cell migration, growth and survival [9,10], as well as being implicated in cancer progression and virus entry [11,12]. The increased expression of CXCR4 has been observed in breast cancer [13], prostate cancer [14] and melanoma [15], as well as in the tumour microenvironment, promoting metastasis, angiogenesis and tumour growth [12,16,17]. CXCR4 is one of the cell surface receptors used for HIV entry into cells, and mutations in its cytoplasmic tail are linked to WHIM syndrome due to its suggested role in the development of the B-lymphocyte cell lineage [18,19]. At a mechanistic level, CXCR4 mediates its effects through the activation of the G⍺_i_ family of G proteins to inhibit adenylyl cyclase, reduce cyclic AMP (cAMP) production, and regulate downstream pathways [7,9]. It exists in the membrane in both monomeric and oligomeric forms, which reorganise in response to agonist (e.g., CXCL12) and antagonist (e.g., IT1t) binding [20,21], and is internalised through clathrin-dependent pathways following phosphorylation by G protein-coupled receptor kinases and the binding of β-arrestin [22]. GPCR plasma membrane organisation therefore has significant implications for receptor function and trafficking [23,24,25], and further investigation of the dynamics of CXCR4 in the plasma membrane will provide insight into the biological processes that precede receptor internalisation.

FFS techniques provide real-time information in a diffraction-limited detection volume, allowing high spatiotemporal resolution within live cells [26,27]. The diffusion coefficients, number (concentration) and molecular brightness of fluorescent species can all be extracted, allowing understanding of species size, environment, stoichiometry, interactions, and the dynamics of these processes. Recent advances in microscope and detector technology have substantially improved the accessibility of FFS techniques such that, on a standard confocal microscope with suitable detectors, several FFS techniques such as Fluorescence Correlation Spectroscopy (FCS), Photon Counting Histogram analysis (PCH), Raster Image Correlation (RICS), and Number and Brightness (N&B) analysis can be used in concert, along with related techniques such as Fluorescence Recovery After Photobleaching (FRAP). Each of these approaches provides complementary information across different temporal and spatial ranges.

**Figure 1:**
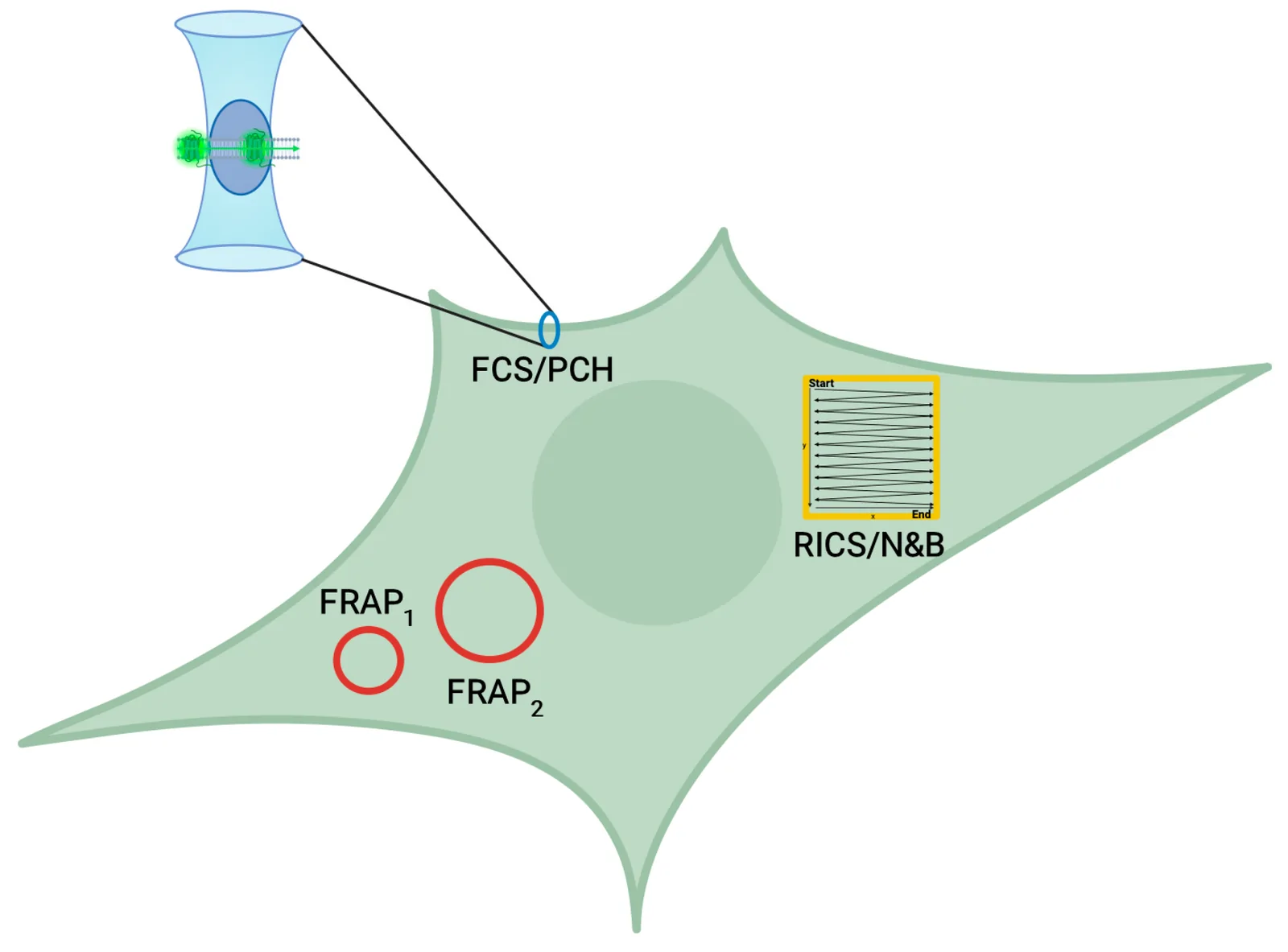
Schematic representation of the detection sizes of the different fluorescence fluctuation spectroscopy techniques used in this study. The detection area of FCS on the membrane is approximately 0.09 µm^2^, whilst FRAP_1_ (20-pixel diameter) and FRAP_2_ (30-pixel diameter) use bleaching areas of 5.87 µm^2^ and 13.22 µm^2^, respectively. The RICS/N&B ROI is 14.38 μm^2^. Created in Biorender. Karsai, N (2026) www.BioRender.com/359mrxd (accessed on 1 May 2026).

Fluorescence Correlation Spectroscopy (FCS) uses autocorrelation analyses of the fluctuations in fluorescence intensity as a fluorescent species moves through a stationary diffraction-limited volume to derive the average species diffusion coefficient (D_FCS_, μm^2^s^−1^) and average particle number (N, μm^−2^) on the nanoscale (~0.09 μm^2^, Figure 1) [28,29]. FCS can also distinguish multiple diffusing species within the same measurement if their dwell times in the detection volume differ significantly (approximately 6-fold) [30], and is ideally suited towards low expression systems, optimally resolving particle numbers of 10–1000 μm^−2^. An alternative analysis of the same fluctuation data, Photon Counting Histogram Analysis (PCH), determines the fluorescence intensity of each species, described by its molecular brightness (ε, count/molecule·s^−1^). Here, the intensity of the fluctuation traces is analysed in small time bins (chosen to be shorter than the dwell time of the species being studied, e.g., 100 µs for membrane proteins with 10 ms dwell time) in order to construct a histogram whose super-Poissonian behaviour can be modelled to extract the average molecular brightness of the species (ε) [31]. As with FCS, PCH analysis can distinguish multiple species, but this time with differing brightness (e.g., oligomers), where they differ by at least 2-fold [32]. PCH is therefore useful for monitoring the stoichiometry of complexes in a similar spatial and temporal resolution to FCS, even when no significant differences in diffusion coefficients are detected [33,34].

Raster Image Correlation Spectroscopy (RICS), like FCS, is used to derive diffusion coefficient (D_RICS_, μm^2^s^−1^) and particle number (N, μm^−2^), but via a line-by-line (raster) scan across a larger area (e.g., 14 µm^2^, Figure 1) rather than a single stationary volume [35,36]. RICS provides an ensemble value of N and D across the whole region of interest, rather than at a single point. Due to this (and the relative total number of photons collected within each spatial unit), RICS works best when resolving single species, although multiple components can be fitted to the resulting autocorrelation curves. RICS autocorrelation curves are modelled along both x and y vectors of the region of interest and can provide a spatial diffusion map if the sampling area is sufficiently large. When creating these diffusion maps, the spatial resolution has been approximated at ~0.8 µm, limited by a 16-pixel analysis unit [37,38]. As with PCH, the same raw data collected in RICS can be used for Number and Brightness Analysis (N&B). N&B is a moment analysis, which uses pixel mean fluorescence intensity and variance to derive the apparent species number and apparent species brightness per pixel of the region of interest [39]. However, unlike PCH, N&B derives an ensemble average, thus multiple species of different brightness cannot be resolved within the observation volume/pixel. Nevertheless, as a whole image region is analysed, a map of the apparent number and brightness of each pixel can be generated, and the macro-spatial organisation of a species examined. If calibrated with a sample of known brightness, the actual molecular brightness and concentration of the species of interest can be calculated [33].

An alternative approach to determining the diffusion coefficient on the microscale is Fluorescence Recovery After Photobleaching (FRAP), which records the intensity of a region of interest over time, before and after a bleaching step. Modelling the recovery of fluorescence over time provides the average diffusion coefficient of the species within the region (D_FRAP_, μm^2^s^−1^) [40]. Whilst spatially it can sample the same dimensions as RICS/N&B, FRAP is able to determine a non-diffusing immobile fraction that is invisible to FFS approaches, which require fluctuations in intensity. In FRAP, the immobile fraction, which is bleached and does not recover over the time course of imaging, can be quantified alongside the mobile fraction (mobile = total − immobile). Whilst defined as the immobile fraction, this also includes very slow-moving species and/or species whose movement is confined within the area being bleached [41,42].

Here, we have used an N-terminal SNAP-tagged CXCR4, and applied four FFS techniques, in combination with FRAP, to derive the diffusion coefficient, particle number and molecular brightness of the receptor species following stimulation with its endogenous agonist, CXCL12, or the small molecule inverse agonist, IT1t. Employing consistent experimental conditions of cell labelling and treatment, and employing all techniques on the same microscope (Zeiss LSM 880; Carl Zeiss, Jena, Germany), we were able to build up a more comprehensive understanding of the ligand-mediated changes in membrane organisation of CXCR4 not accessible to standard imaging approaches.

## 2. Materials and Methods

### 2.1. Cell Culture

The HEK293G SNAP-CXCR4 clonal stable cell line, stably expressing an N-terminal SNAP-tagged CXCR4 receptor (SNAP-CXCR4) as described by Dekker et al. [43], with the G-suffix indicating the presence of the GloSensor cAMP biosensor [44] (Promega Corporation, Madison, WI, USA), was cultured in Dulbecco’s modified Eagle’s medium (DMEM; D6429, Sigma-Aldrich, Darmstadt, Germany), supplemented with 10% Foetal Bovine Serum (FBS; Sigma-Aldrich).

### 2.2. GloSensor Assay

Cells were plated at 3 × 10^4^ cells/well in white 96-well clear bottom multiwell plates pre-coated with poly-D-lysine (10 µg/mL) in growth media 24 h prior to the experiment and maintained at 37 °C, 5% CO_2_. On the day of the experiment, the media was aspirated and replaced with 80 µL of warmed HEPES-buffered saline solution (HBSS: pH 7.45; Sodium pyruvate, 2 mM; NaCl, 145 mM; D-Glucose, 10 mM; KCl, 5 mM; MgSO_4_.7H_2_O, 1 mM; HEPES, 10 mM; CaCl_2_ 1.3 mM; NaHCO_3_ 1.5 mM) containing GloSensor™ cAMP reagent (6%) (Promega) with or without AMD3100 (Sigma-Aldrich; 100 nM–10 µM) and incubated for 2 h at 37 °C. Luminescence was recorded on an EnVision Multilabel Plate Reader (Perkin Elmer, Shelton, CT, USA) continuously over 60 min following the simultaneous addition of 30 µM Forskolin (Sigma-Aldrich) and CXCL12 (recombinant Human SDF-1α, PeproTech, Cranbury, NJ, USA; 1 fM–100 nM).

### 2.3. Labelling of SNAP-CXCR4

Cells were plated at 1.5 × 10^4^ cells/well in 8-well Nunc™ Lab-Tek™ chambered coverglasses (155361, no. 1.0 borosilicate glass) pre-coated with poly-D-lysine (10 µg/mL) in growth media 48 h prior to the experiment and maintained at 37 °C in a humidified atmosphere of 5% CO_2_. On the day of the experiment, the cells were incubated with 0.1 µM SNAP-Surface™ AlexaFluor™488 (membrane impermeable) (New England Biolabs, Ipswich, MA, USA) in growth media for 30 min at 37 °C in 5% CO_2_. The SNAP system consists of a 20 kDa O^6^-alkylguanine-DNA alkyltransferase, which reacts with benzylguanine (BG) derivatives carrying a fluorescent tag [45] in our experiments, irreversibly labelling the SNAP-CXCR4 with an AlexaFluor™488 label. Cells were then washed three times in warmed HBSS over the course of 20 min, before being left in a final volume of 200 µL HBSS. Cells were either pre-incubated with vehicle (HBSS), 1 µM IT1t (30 min; N,N′-dicyclohexylcarbamimidothioic acid (5,6-dihydro-6,6-dimethylimidazo[2,1-b]thiazol-3-yl)methyl ester, Tocris Bioscience, Bristol, UK) or 10 nM CXCL12 (10 min) at 37 °C before being allowed to equilibrate to 24 °C and then used as detailed below.

### 2.4. Microscope Set-Up

All experiments were performed at 24 °C on a Zeiss LSM 880 confocal microscope on a Zeiss Axio Observer Z1 stand (Carl Zeiss, Jena, Germany) with a 488 nm Argon laser using a 40× c-Apochromat 1.2 NA water immersion objective. The precise beam-path, pinhole, laser power and laser scan speed varied depending on the modality, as detailed below. Gain and offset were kept consistent within an experiment, unless stated otherwise. Image and data analyses for all methodologies were performed using Zen Black (2012) (Carl Zeiss).

#### 2.4.1. Confocal Imaging

Single equatorial confocal images were captured using the 488 nm argon laser (~0.7 kW/cm^2^) and a 505–610 nm emission filter. The pinhole was set at 1 Airy Unit (AU) and images were collected at 1024 × 1024 pixels (69 nm/pixel) with a scan speed of 2.05 μs/pixel.

#### 2.4.2. Fluorescence Correlation Spectroscopy (FCS) and Photon Counting Histogram (PCH) Analysis

**Figure 2:**
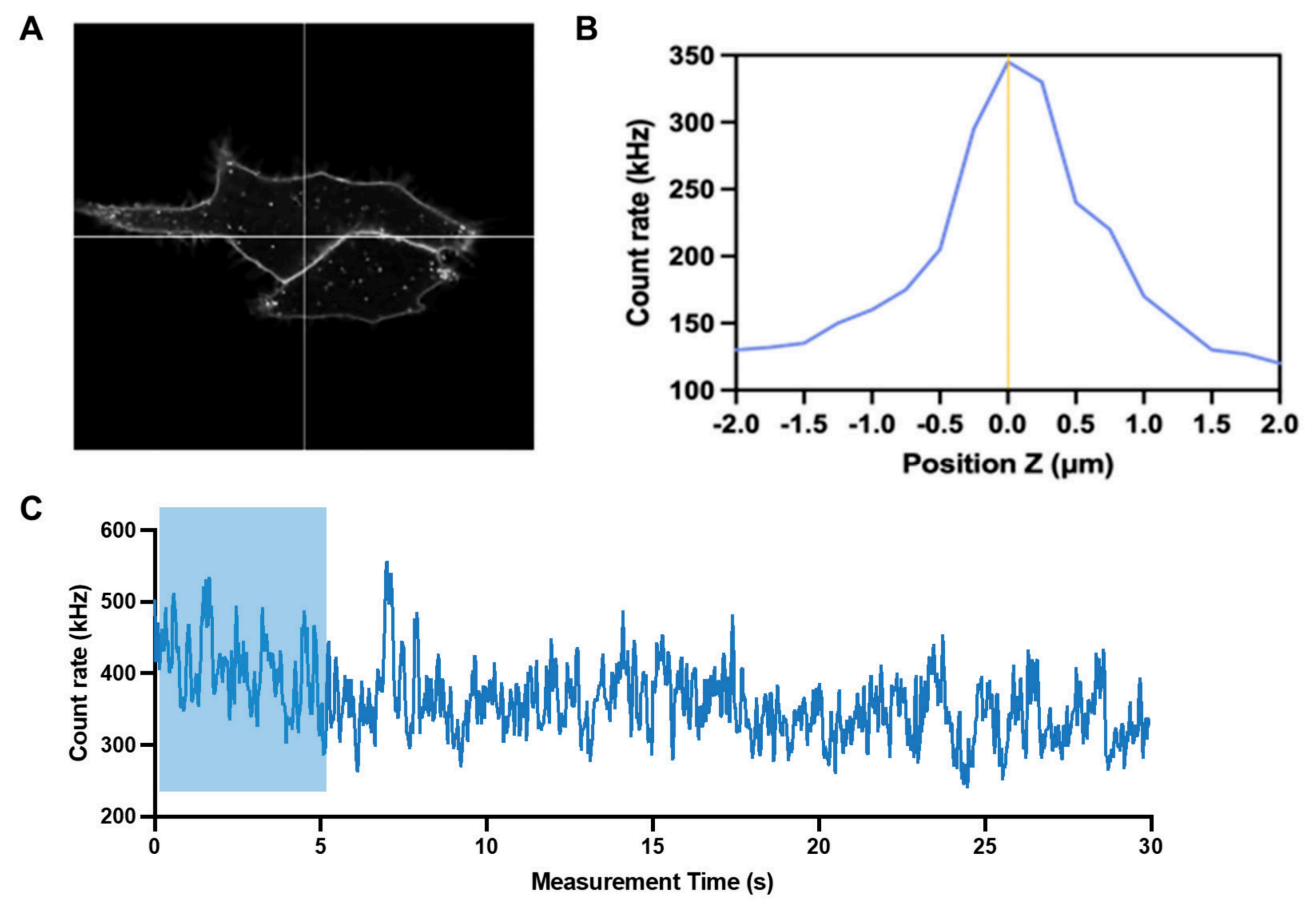
Cell-based FCS. (**A**) The detection volume is positioned in x-y via standard confocal imaging. (**B**) The detection volume is precisely positioned in z, using an automated z intensity scan, where the peak (yellow line) represents the upper cell membrane. (**C**) Representative example of the fluctuation trace of the cell membrane read obtained from the upper membrane of an HEK293G SNAP-CXCR4 cell labelled with SNAP Surface™ AlexaFluor™488. The first 5 s of the trace are routinely omitted from analysis to exclude the initial bleaching effects of immobile species (blue shading).

For FCS readings, the detection volume was initially positioned using a live confocal image (Figure 2A; 512 × 512 pixels, 137 nm/pixel) and then precise z-position achieved following an intensity z-scan ±2 µm, with 0.25 µm interval (Figure 2B), as described in Goulding et al. (2021) [46]. The detection volume (0.17–0.27 fL, theoretical membrane area of 0.09 μm^2^) was positioned on the apical plasma membrane with the 488 nm argon laser (~0.04 kW/cm^2^). Single point fluctuation traces were then recorded for 30 s per cell, with emission collected through a 508–691 nm bandpass filter and the pinhole set to 1 AU. The lateral and axial radii of the detection volume were determined on each experimental day by measuring the dwell time of 20 nM ATTO488 (Sigma-Aldrich); using the literature diffusion coefficient 400 µm^2^s^−1^, [47], using the equations

(1) $$D=\frac{\omega_{r}^{2}}{4\;\cdot \;\tau_{Di}}$$

(2) $$V=\pi^{3/2}\cdot \omega_{r}^{2}\cdot \omega_{a}$$

where D is the diffusion coefficient of a fluorescent species with a dwell time of τ_Di_ in a confocal detection volume, V, with lateral radius, ω_r_, and vertical half height of ω_a_.

Autocorrelation analysis was performed on fluctuation traces within Zen Black (2012) and curves fitted to a 2-component diffusion model comprising a single 3D species, describing free SNAP Surface^TM^ AlexaFluor™488, with dwell time constrained to 20–80 µs, and a single 2D species, describing the membrane-restricted movement of SNAP-CXCR4 (Supplementary Figure S1) [46]. The autocorrelation function (G(τ)) of the curve, which describes the diffusion of the fluorescent species through a 3D Gaussian detection volume, can be described as

(3) $$G\left(\tau \right)=1+\frac{A}{N}\cdot \sum_{i=1}^{m}\cdot f_{i}\cdot {\left(1+\frac{\tau }{\tau_{D_{i}}}\right)}^{-1}\cdot {\left(1+\frac{\tau }{S^{2}\cdot \tau_{D_{i}}}\right)}^{-\frac{1}{2}}$$

where A is a pre-exponential term to account for fluorophore photo-physics, defined by

(4) $$A=1+\frac{T}{1-T}e^{-\tau /\tau_{T}}$$

G(τ) is the normalised intensity autocorrelation function with an average of N fluorescent particles with a dwell time of τ_Di_. f_i_ is the fractional contribution of the species, i, of a total m species, to the autocorrelation amplitude. S describes the structural parameter, a ratio of the vertical to axial radius of the detection volume, which was fixed at 5. Whilst imperative for determining the daily confocal volume dimensions, variations in S have only a minor effect on derived parameters, so fixing it at 5 is commonplace. A is a pre-exponential factor that describes the contribution to the signal from fluorophore photo-physics, where T is the percentage of molecules in the triplet state and τ_T_ is its lifetime, which was constrained to <10 μs. For the 2D component, describing the diffusion of a membrane restricted receptor, which is confined to the 2D plane, the structural parameter, S, approaches ∞, and the equation simplifies to

(5) $$G(\tau )=1+\frac{A}{N}\cdot \sum_{i=1}^{m}\cdot f_{i}\cdot {\left(1+\frac{\tau }{\tau_{D_{i}}}\right)}^{-1}$$

The first 5 s of the traces were routinely discarded to account for the initial bleaching of immobile fluorescent species (Figure 2C) and the curves fit with a combination of Equations (3) and (5). Only those curves in which the asymptote was clearly defined were included in the data analysis.

**Figure 3:**
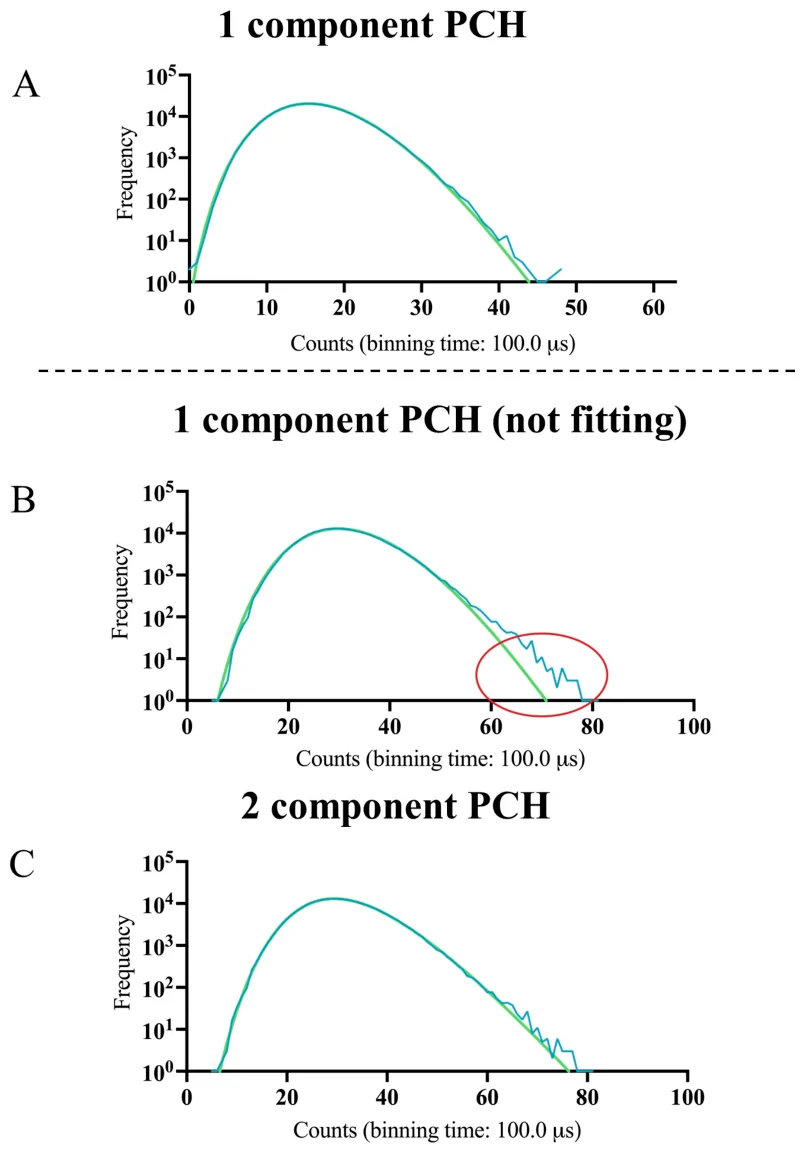
PCH analysis. (**A**) 1-component PCH fit (green line) of the representative data trace (blue line). The binning time was set at 100 µs. (**B**) Representative data that presented a significant deviation from the fit at high photons per bin (red circle), so a second component was added to the PCH fit (**C**).

Fluctuation traces were also analysed by PCH analysis. PCH analysis derives the average molecular brightness and particle number of the fluorescent species from a fluctuation trace via the fit deviation between a super-Poissonian and a simple Poissonian model applied to a histogram constructed from the time-binned fluctuation trace [31]. Traces were analysed within Zen Black (2012) using a 3D PCH model, and the time bin was set at 100 μs, appropriate for membrane-restricted receptors, with the first-order correction constant determined from a daily calibration with 20 nM ATTO 488. Histograms were routinely modelled with 1-component PCH analysis (Figure 3A). In cases where there was a significant deviation from the fit at high photons per bin, a 2-component PCH analysis was used (Figure 3B,C).

#### 2.4.3. Fluorescence Recovery After Photobleaching (FRAP)

**Figure 4:**
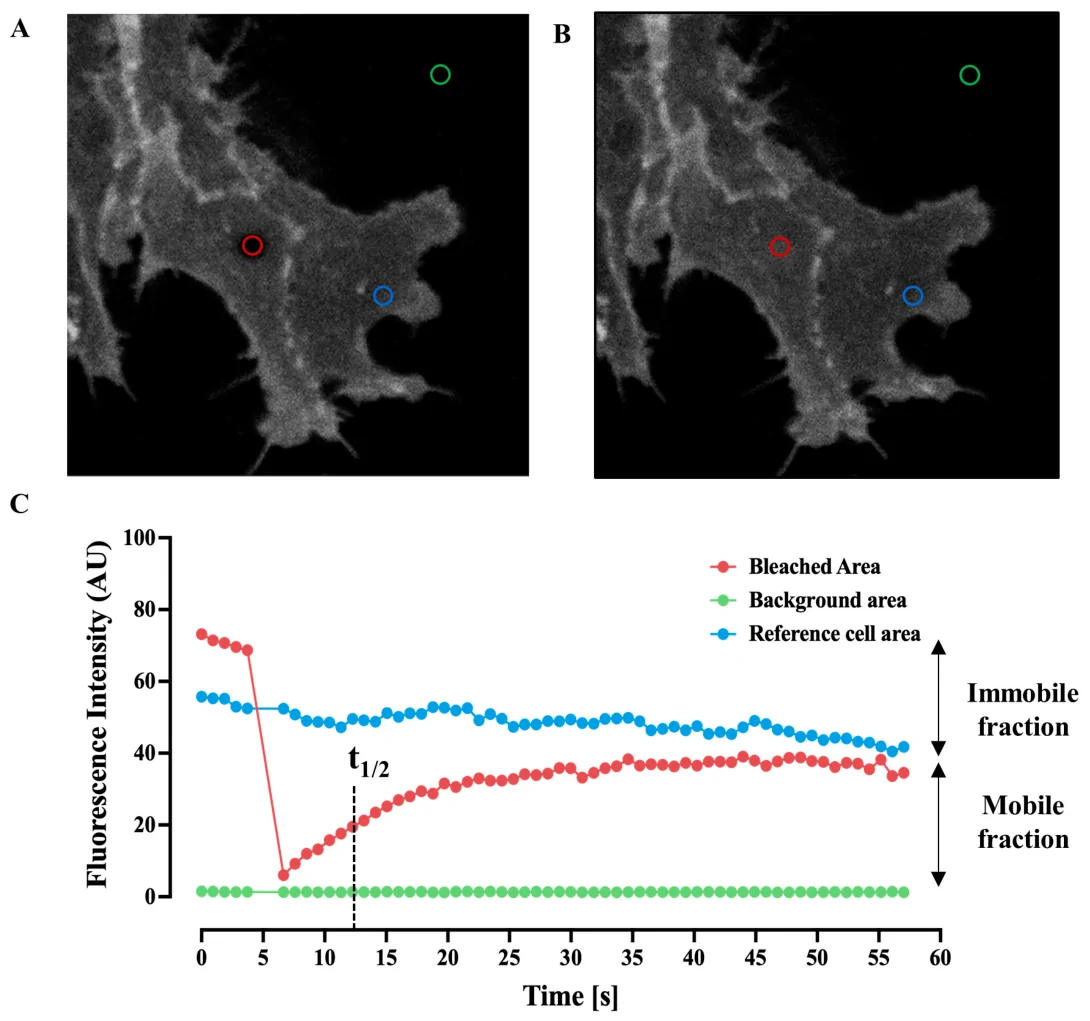
FRAP analysis. (**A**) Representative images of the basal membrane of the cell, directly after bleaching step (**B**) or following recovery. The red circle indicates the bleached area, the green circle is the background correction reference and the blue circle is the non-bleached reference area. (**C**) Representative FRAP curve (red) recording the fluorescence intensity change over time alongside reference and background traces. The immobile fraction, mobile fraction and t_1/2_ (time taken for recovery of half the fluorescence intensity) for the region of interest (red) are depicted.

For FRAP measurements, confocal imaging was used to focus on the basal membrane of HEK293G SNAP-CXCR4-expressing cells. Sixty consecutive confocal images were collected of a 512 × 512 pixel region with a 50-bleach iteration at 100% laser power after 5 scans (circular bleach spot of variable size 5.89–13.26 µm^2^). Scans were made with the 488 nm Argon laser (~0.7 kW/cm^2^), with emission collected through a 500–550 nm bandpass emission filter, detected by the GaAsP detector, used in integration mode. The pinhole was set to 1.5 AU, with a pixel dwell time of 1.52 µs and pixel size set to 137 nm. The change in the average intensity of the circular bleach spot was plotted against time, following background correction and normalisation to a reference area from a cell within the same image capture, to account for bleaching caused by scanning. An exponential recovery curve was fitted, from which the half time of recovery (τ_1/2_) was determined using the equation [48]

(6) $$I=IE-I1\cdot e^{\left(-\tau /T1\right)}\;$$

where I is intensity, I1 is the mobile fraction intensity and IE is the final signal intensity. T1 is the fitted parameter of time of recovery. I1 was determined as the percentage of the intensity of the recovery plateau compared to the pre-bleached intensity. The immobile fraction could subsequently be calculated from the difference between the plateau intensity and the pre-bleached intensity (Figure 4C). The diffusion coefficient (D_FRAP_) of a circular bleach area was calculated via the equation

(7) $$D_{FRAP}=\frac{\omega_{b}^{2}}{4\cdot (\tau_{1/2})}$$

where ω_b_ (μm) is the radius of the bleached area.

#### 2.4.4. Raster Image Correlation Spectroscopy (RICS) and Number & Brightness (N&B)

A 100-frame image series was captured on the basal membrane of the cell with the 488 nm argon laser set at 0.3% excitation (~0.1 kW/cm^2^) and emission collected through a 500–550 nm emission filter, with the pinhole set to 1 AU. The GaAsP detector was used in photon-counting mode with a PC gain (detector gain) of 0.2. The capture field of view was set at 256 × 256 pixels with a pixel size of 50 nm and a pixel dwell time of 8.24 µs. A region of interest (ROI) of 80 × 80 pixels within this field of view, devoid of obvious cell structures to faithfully represent the basal membrane, was selected for analysis.

Image series were initially analysed using the RICS module in Zen Black (2012) to construct autocorrelation curves in both x and y. Out-of-focus frames due to cell movement were excluded from the image series with a minimum of 50 continuous frames set as the criterion for inclusion in the dataset. A rolling 5-frame average was allowed to remove slow moving structures from the analysis. Autocorrelation curves were fit with a single component 2D model using the equation

(8) $$G\left(\xi ,\psi \right)=F+\frac{\gamma }{N}\cdot G_{S\;}\cdot G_{d}$$

where G(ξ,ψ) is the autocorrelation function for diffusion in the x and y directions. F is an offset to allow for cell movement, γ is a geometric correction factor for the Point Spread Function (PSF), which was set at 1, modelling the PSF as cylindrical, and N is the number of fluorescent particles. Gs is the scanning term of the equation and Gd the translational diffusion term, where

(9) $$Gs(\xi ,\psi )=e^{-\left(\frac{{\left(\frac{\xi \cdot \delta_{r}}{\omega_{r}}\right)}^{2}+{\left(\frac{\psi \cdot \delta_{r}}{\omega_{r}}\right)}^{2}}{\left(1+\frac{\xi \cdot \tau_{p}+\psi \cdot \tau_{l}}{\tau_{d}}\right)}\right)}$$

and

(10) $$Gd(\tau )=\frac{1}{N}\cdot {\left(1+\frac{\tau }{\tau_{D_{i}}}\right)}^{-1}$$

Here, ξ and ψ are the correlation coordinates for x and y, respectively, δ_r_ is the pixel size, ω_r_ is the lateral focus radius, τ_p_ is the pixel time, τ_l_ is the line time and τ_d_ is the diffusion time. ω_r_ was determined from the daily calibration with ATTO488 (Equation (1)). From the correlation, the diffusion coefficient (D_RICS_) was calculated within the RICS analysis module as follows

(11) $$D_{RICS}=\frac{\omega_{r}^{2}}{4\cdot t_{d}}$$

Only those curves in which the asymptote was clearly defined were included in the data analysis.

The same image series data were then reanalysed for Number and Brightness (N&B) analysis in Zen Black (2012). A de-trending filter of 10 frames was applied to correct for bleaching and cell movement. A minimum threshold for intensity values was set at 0.01. N&B analysis calculates the apparent number of particles and brightness per pixel from the measured intensity fluctuations via the following equations

(12) $$B=\frac{\sigma^{2}-\sigma_{0}^{2}}{\langle k\rangle -offset}$$

(13) $$N=\frac{{\left(\langle k\rangle -offset\right)}^{2}}{\sigma^{2}-\sigma_{0}^{2}}$$

Here, B is apparent brightness and N is apparent particle number, σ^2^ is signal variance and $\sigma_{0}^{2}$ readout noise due to the microscope. $\langle k\rangle$ is the mean signal and an offset is allowed to account for dark signal from the detectors, set at 0 for GaAsP detectors in photon-counting mode. An ROI of 12 μm^2^, devoid of obvious cell structures to faithfully represent basal membrane, was selected to extract the apparent number and brightness of SNAP-CXCR4 expressed on the cell membrane.

### 2.5. Statistical Analysis

The data were imported into GraphPad Prism Version 10 (GraphPad Software, San Diego, CA, USA) to generate the figures and, where applicable, assess statistical significance. The ‘*n*’ represents measurements from individual cells per condition from a minimum of 3 independent biological replicates. One-way ANOVA followed by post hoc Tukey’s multiple comparison test [49] was carried out to compare ligand-induced conditions against basal conditions, with statistical significance defined as *p* < 0.05. In the case of FRAP data, two-way ANOVA followed by Šídák’s multiple comparisons test [50] was applied to compare multiple ligand additions in different bleaching sizes, with statistical significance defined as *p* < 0.05.

## 3. Results

### 3.1. Functional SNAP-CXCR4 Is Expressed at the Membrane of HEK293G Cells

**Figure 5:**
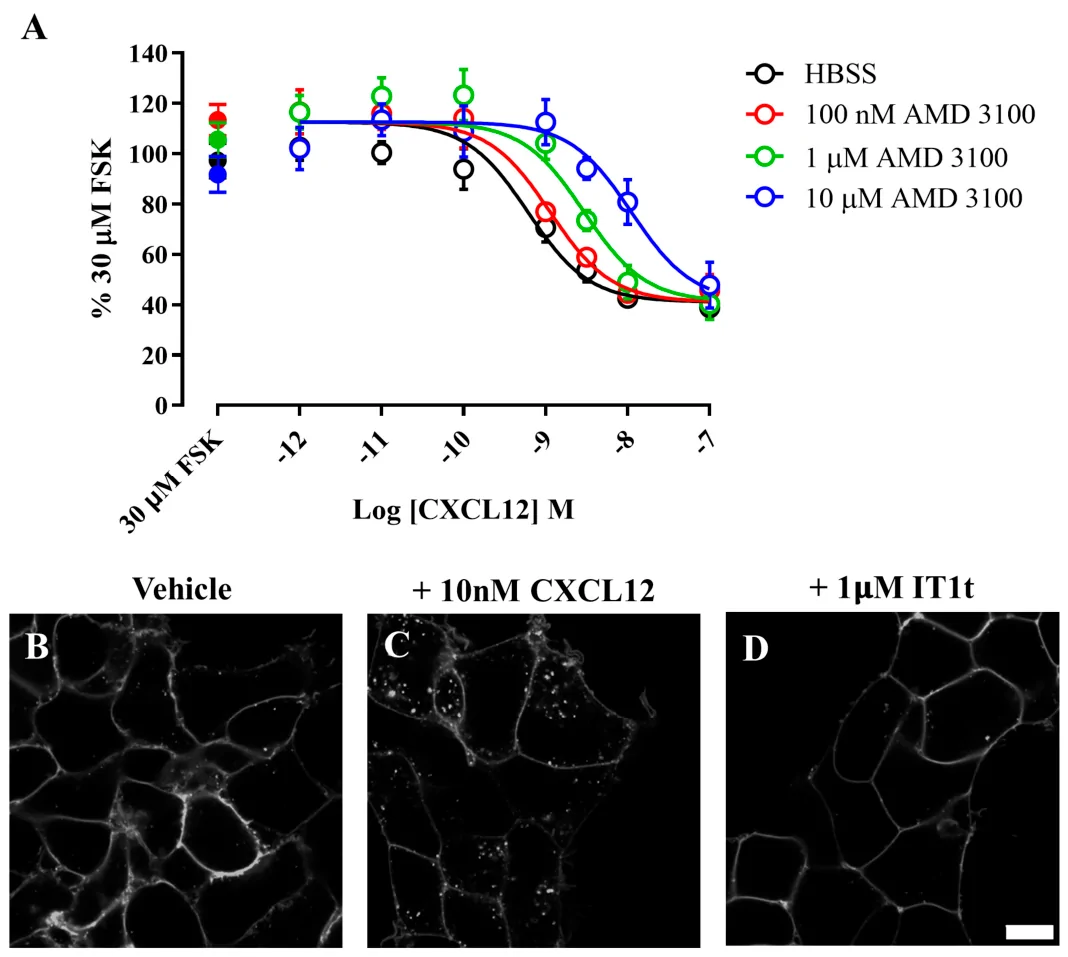
Spatial distribution of SNAP-CXCR4. (**A**) The CXCL12-mediated inhibition of forskolin (FSK)-stimulated cAMP response in the presence and absence of AMD3100 (100 nM–10 μM). Responses are normalised to that of the response seen with 30 µM forskolin alone. Data are mean ± S.E.M. of pooled data from 5 independent experiments. (**B**–**D**) Equatorial confocal images of HEK293G SNAP-CXCR4 cells labelled with 0.1 µM SNAP-surface AlexaFluor™488. Cells were treated with (**B**) vehicle, (**C**) CXCL12 (10 nM, 10 min, 37 °C) or (**D**) IT1t (1 µM, 30 min, 37 °C). Scale bar shows 10 µm, with images representative of three independent experiments performed.

To investigate changes in the membrane organisation of CXCR4, in response to agonist and inverse agonist ligands, we used a clonal HEK293G SNAP-CXCR4 cell line stably expressing an N terminal labeled SNAP-CXCR4 and the Glosensor cAMP biosensor [44]. Initially, SNAP-CXCR4 expression and function were confirmed. CXCL12 stimulation elicited a concentration-dependent inhibition of forskolin-stimulated cAMP production with a pEC_50_ of 9.22 ± 0.14 (*n* = 5). This response could be competitively antagonised by pre-incubation with the CXCR4 antagonist AMD3100 (Figure 5A) with a pA_2_ of 6.90 ± 0.45 (*n* = 5), consistent with its affinity at CXCR4 [51], and this confirmed CXCR4’s agonist and antagonist pharmacology, as expected. The pA_2_ value is a measure of the affinity of a competitive antagonist and can be derived by measuring the shift in response to an agonist following increasing concentrations of antagonist. Following labelling with the membrane impermeant SNAP-Surface™ AlexaFluor™488, SNAP-CXCR4 localisation was observed with standard confocal microscopy. In unstimulated conditions, SNAP-CXCR4 was localised to the cell membrane (Figure 5B). Upon agonist stimulation (10 nM CXCL12, 10 min, 37 °C), multiple punctate cytosolic vesicles were observed, consistent with the agonist-mediated internalisation of CXCR4 (Figure 5C). In contrast, following treatment with the small molecule inverse agonist IT1t (1 µM, 30 min, 37 °C), CXCR4 distribution was comparable to that seen in the vehicle-treated cells, with the majority of SNAP-CXCR4 localised to the cell membrane (Figure 5D).

### 3.2. Fluorescence Correlation Spectroscopy (FCS)

**Figure 6:**
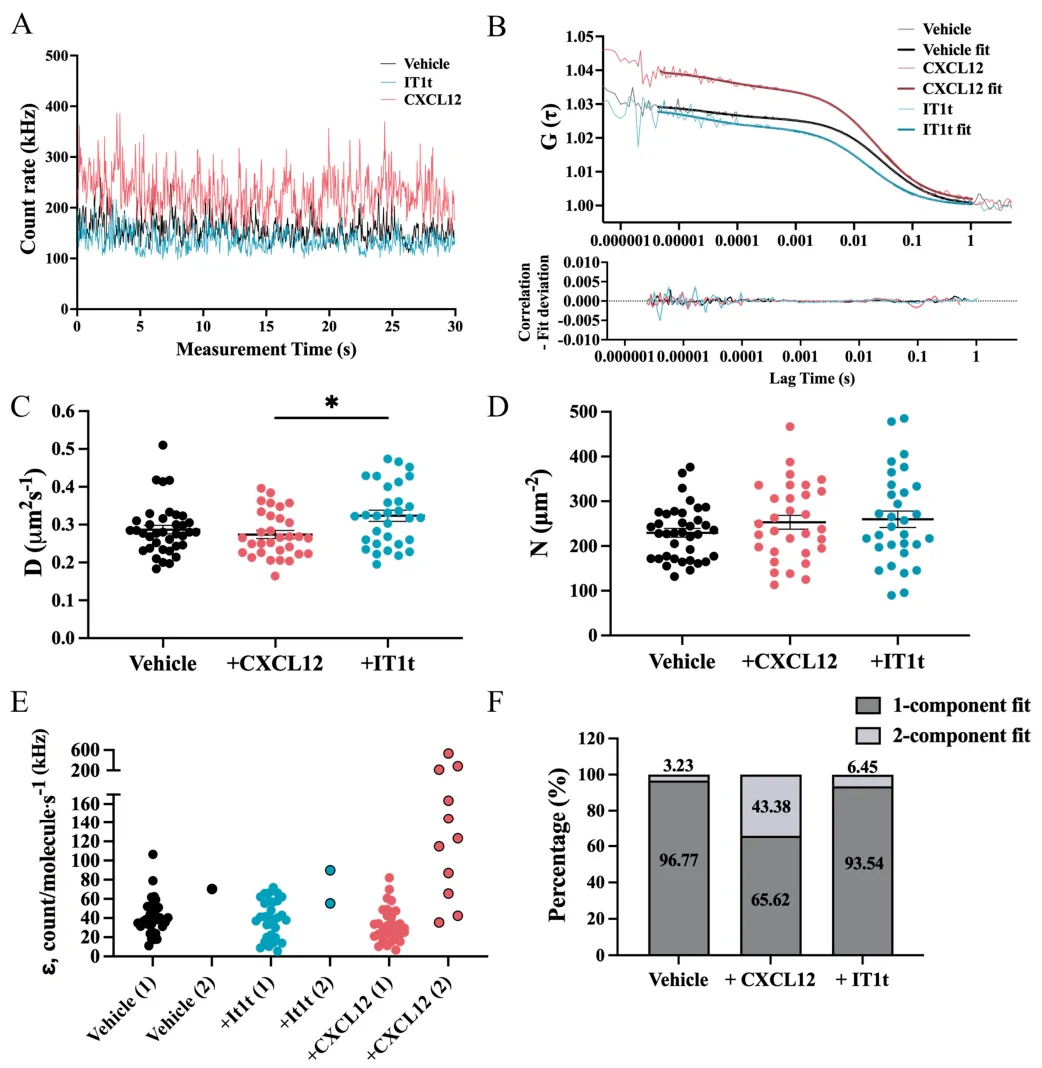
Diffusion coefficient, particle number and molecular brightness as derived from FCS and PCH analysis. (**A**) Representative fluctuation traces following vehicle (black), CXCL12 (red) and IT1t (blue) addition. (**B**) Representative autocorrelation curves (thin lines) and diffusion model fit (thick lines) with deviation of raw data from fit displayed below. Extracted diffusion coefficient (**C**), particle number (**D**) and molecular brightness (**E**,**F**) following treatment with either vehicle, CXCL12 or IT1t (*n* = 31–37 cells from a minimum of 6 independent experiments). Data displayed are separate cell reads with mean ± SEM. (**F**) Percentage of cells that preferred a 1- or 2-component brightness fit following PCH analysis. Significance tested with one-way ANOVA followed by Tukey’s multiple comparisons test (* *p* < 0.05).

Having demonstrated the functional integrity of SNAP-CXCR4 when stably expressed in the HEK293G cells, and confirmed its distribution at a single cell level, we used FCS to investigate the diffusive characteristics of the receptor at the nanoscale. The FCS detection volume was positioned on the apical membrane of the SNAP-Surface™ AlexaFluor™488-labelled HEK293G SNAP-CXCR4 cells pre-incubated in HBSS in the presence or absence of CXCL12 (10 nM, 10 min, 37 °C) or IT1t (1 μM, 30 min, 37 °C). A sub-maximal agonist dose (10 nM) and short duration of treatment (10 min, 37 °C) ensured the stimulation of a ligand-driven effect, whilst also maintaining sufficient membrane expression to capture receptor reorganisation prior to extensive internalisation. A saturating concentration of SNAP-Surface™ AlexaFluo™488 (100 nM) was used to ensure maximal labelling [52]. Fluctuation traces (1 × 30s) were collected, analysed by autocorrelation analysis and subsequently fitted in Zen Black (2012) (Figure 6A,B). As in our previous studies of other SNAP-tagged GPCRs, autocorrelation curves required a two-component diffusion model for an acceptable fit [46,52] (Supplementary Figure S1). This model comprised a faster 3D component representing free, unbound SNAP-Surface™ AlexaFluor™488 dye (14.6 ± 0.5% of the AC curve amplitude) and a slower component representing the labelled SNAP-CXCR4 on the cell membrane (see Section 2.4.2). Under basal conditions, the diffusion coefficient (D_FCS_) of SNAP-CXCR4 was 0.29 ± 0.011 μm^2^s^−1^ (Table 1; *n* = 37 cells from 10 independent experiments). Whilst the diffusion coefficient following treatment with either ligand was not significantly changed from that observed with the vehicle, CXCL12 and IT1t did have significant opposing effects to each other, with the diffusion coefficient following CXCL12 treatment being signficiantly lower than that following IT1t treatment (Figure 6C; * *p* < 0.05; 10 nM CXCL12—0.27 μm^2^s^−1^ ± 0.011 μm^2^s^−1^, *n* = 31; 1 µM IT1t—0.32 ±0.015 μm^2^s^−1^ *n* = 31 cells from six individual experiments). Neither treatment had a significant effect on particle number (N; Figure 6D, vehicle—230 ± 10 μm^−2^; 10 nM CXCL12—253 ± 15 μm^−2^; 1 µM IT1t—260 ± 18 μm^−2^). It should be noted at this point that in this context, N defines particle number, not receptor number; multiple receptors diffusing as a single species (e.g., oligomers) would present as a single particle.

### 3.3. Photon Counting Histogram (PCH)

Fluctuation traces from FCS experiments were also analysed using PCH analysis to determine the average molecular brightness of the detected fluorescent populations. Under basal conditions, most cells displayed a brightness profile that could be modelled by a single component with a molecular brightness (ε) of 41.34 ± 3.47 kHz count/molecule·s^−1^ (Table 1, Figure 6E,F; *n* = 31 cells from eight independent experiments). Only one cell required a 2-component PCH fit, the first component (dimmer) displaying a brightness (ε = 31.25 kHz) in line with the 1-component fit species and the second describing a species with 2.3-fold greater molecular brightness (ε = 70.30 kHz). Interestingly, whilst the first component population observed following CXCL12 treatment was similar in brightness (33.14 ± 3.13 kHz count/molecule·s^−1^, *n* = 31 from six individual experiments) to that of vehicle-treated cells, the percentage of cells that displayed a 2-component brightness profile increased to 34%. This second component brightness accounted for 15.7 ± 7.3% of the species in each trace, and had a much greater range of brightness, namely, 35–600 kHz count/molecule·s^−1^, with an average of 163.9 kHz count/molecule·s^−1^ (Supplementary Table S1). This change is consistent with an increase in CXCR4 oligomerisation and/or clustering in response to agonist. In contrast, a similar profile to that of the vehicle-treated cells was observed following pre-incubation with the inverse agonist IT1t, with most cells displaying a brightness profile modelled by a single component with a mean brightness of 39.26 ± 3.53 kHz (*n* = 31 from six individual experiments), suggesting that IT1t did not induce SNAP-CXCR4 clustering, as it had no detectable effect on the average molecular brightness of the SNAP-CXCR4 population.

### 3.4. Raster Image Correlation Spectroscopy (RICS)

**Figure 7:**
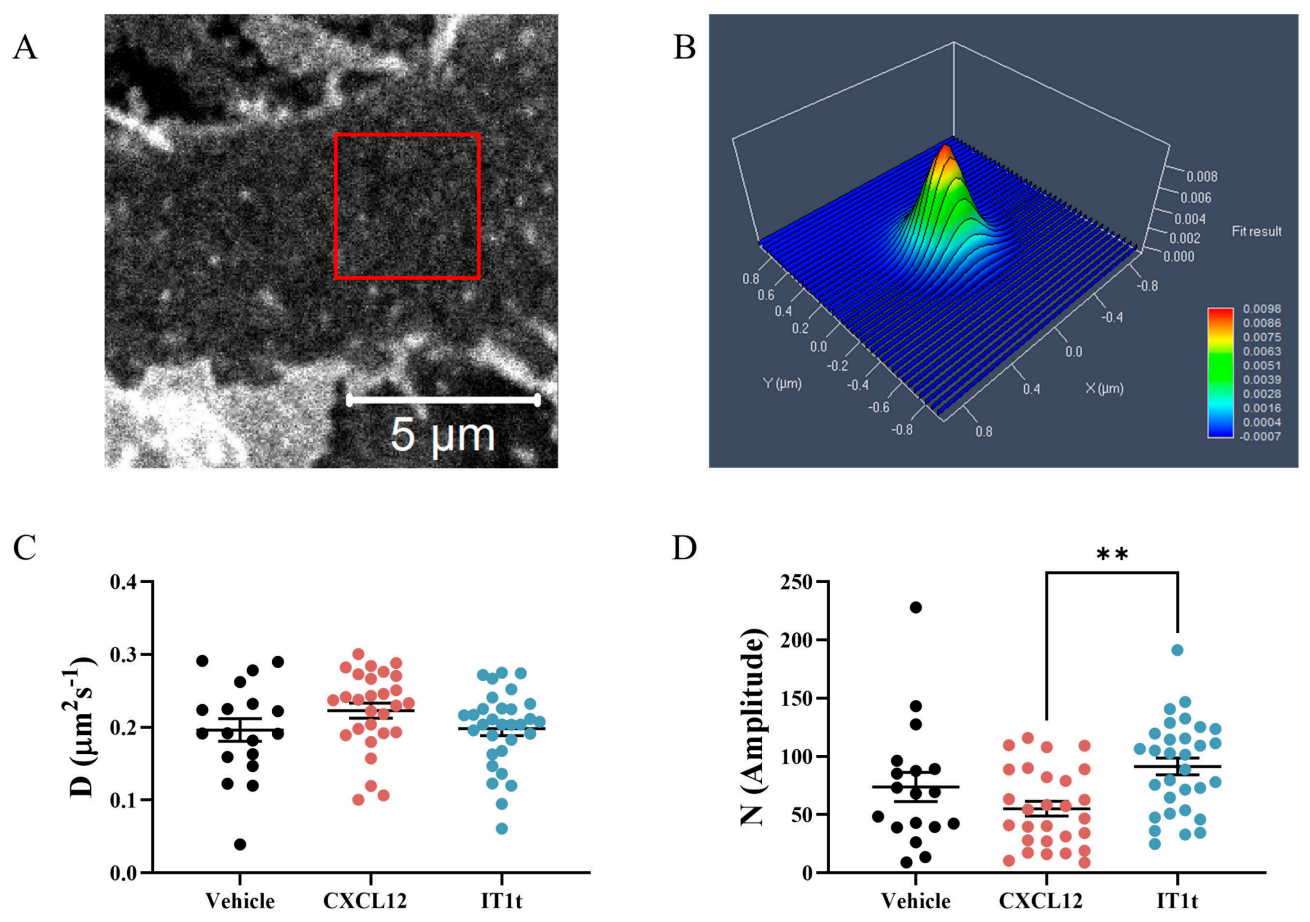
Diffusion coefficient and species amplitude as derived from RICS. (**A**) 256 × 256 pixel field of view of the basement membrane with a subset (red square, 80 × 80 pixel) chosen for analysis. (**B**) A 3D representation of the autocorrelation curves modelled with a 1-component 2D model of diffusion. Derived diffusion coefficient ((**C**) μm^2^s^−1^) and amplitude (**D**) for vehicle (*n* = 18), CXCL12 (*n* = 28) and IT1t (*n* = 31) treatment. Data plotted are individual cell measurements with mean ± S.E.M. Significance tested with one-way ANOVA followed by Tukey’s multiple comparisons test (** *p* < 0.01).

Next, we examined the dynamics of CXCR4 over a larger area employing RICS. RICS provides information on the microscale receptor organisation, minimising any effects of nanoscale heterogeneity that might skew the overall diffusion profile. One hundred scan cycles were taken on the basal membrane of HEK293G SNAP-CXCR4 cells, over an area of 14.38 μm^2^ (80 × 80 pixels) that contained no obvious cell structures, pre-incubated in HBSS in the presence or absence of CXCL12 (10 nM, 10 min, 37 °C) or IT1t (1 μM, 30 min, 37 °C) (Table 2, Figure 7A). Autocorrelation curves were constructed to represent correlated fluorescence movement pixel-to-pixel along the direction of the raster (row, x) and between raster lines (lines, y). These correlation curves were fit to a single 2D component diffusion model (Figure 7B). An average diffusion coefficient for the whole region under basal conditions was determined as 0.20 ± 0.02 µm^2^s^−1^ (*n* = 18 cells from eight individual experiments), slower than that seen in single-point FCS. Consistent with FCS measurements, pre-incubation with neither CXCL12 nor IT1t significantly changed the average diffusion coefficient (0.22 ± 0.01 and 0.20 ± 0.01 µm^2^s^−1^, respectively; Figure 7C; *n* = 28/31 cells from 10 independent experiments). The average species amplitude in RICS measurements, which is related to particle number, was 73.9 ± 12.5 under basal conditions. The particle number derived following CXCL12 treatment was not significantly different from the vehicle condition but was significantly lower than that following IT1t treatment (55.2 ± 6.3 and 91.4 ± 7.1, respectively; Figure 7D; *n* = 28/31 cells *p* < 0.01, vehicle *n* = 18 cells), suggestive of an opposing influence on oligomerisation status.

### 3.5. Number and Brightness (N&B) Analysis

**Figure 8:**
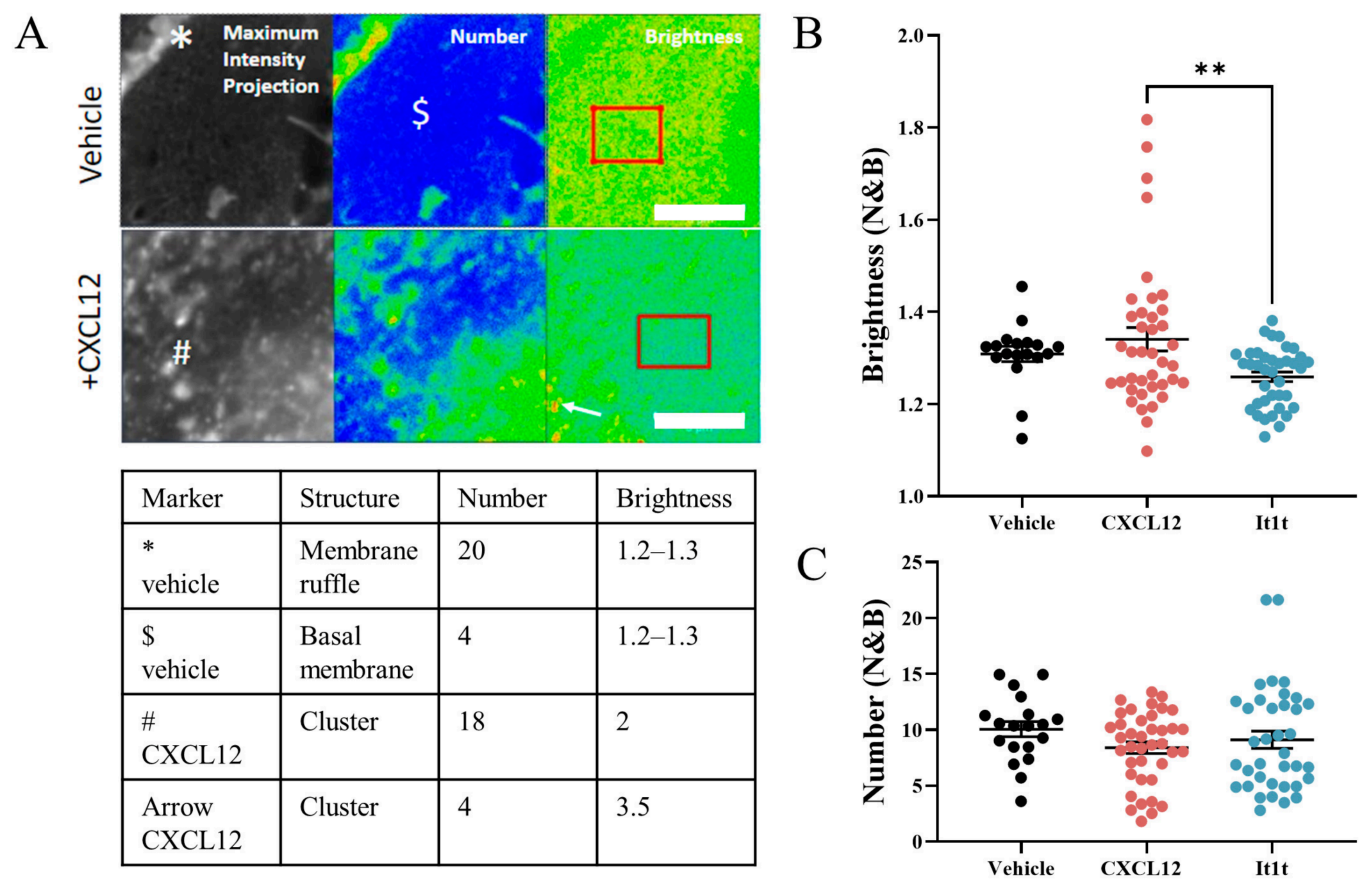
Pixel apparent number and brightness derived from N&B analysis. (**A**) L-R representative maximum intensity projection, number map and brightness map of a 256 × 256 pixel field of view following treatment with vehicle or CXCL12. A 12 μm^2^ region of interest (red rectangle), devoid of obvious cell structures, was selected to derive the average parameters. Scale bar = 5 µm. The table in (**A**) provides illustrative examples of the apparent number and brightness values of cell elements as taken from the single example cells depicted. (**B**) Derived apparent brightness and (**C**) apparent number of fluorescent species from cells treated with vehicle (*n* = 18), CXCL12 (*n* = 38) and IT1t (*n* = 39). Data plotted are individual cell measurements with mean ± S.E.M. One-way ANOVA followed by Tukey’s multiple comparisons test (** *p* < 0.01).

N&B analysis was performed on the same image stacks used for RICS, and three pseudo-colour image maps were constructed per image series within Zen Black (2012). These maps represent the maximum intensity projection, the apparent number image, and the apparent brightness image of the selected image series (Figure 8A). The apparent number represents a singular species and not receptor number. Under basal conditions, cellular structures, e.g., membrane ruffles (Figure 8A *), displayed higher apparent number but lower apparent brightness. While these structures contain SNAP-CXCR4 receptors, our data suggest that each exists as a low-order oligomer. In contrast, bright objects, which are termed clusters, display high brightness with variable apparent number (Figure 8A #, arrow). These objects may reflect large-order oligomers or indeed a collection of receptors associated, for example, with a cellular structure and represented as a single species. To prevent these cell structures and large clusters from distorting the quantification of membrane receptor stoichiometry, a 12 µm^2^ subset region (red rectangle) from each image series was selected for the analysis of number and brightness following ligand treatment. Under basal conditions, the apparent brightness of the membrane appears homogeneous (Table 3, Figure 8B; Apparent brightness = 1.31 ± 0.02; coefficient of variation = 5.33%; *n* = 18 cells from eight independent experiments). In the presence of CXCL12, the population becomes more heterogeneous (1.34 ± 0.02; coefficient of variation = 11.83%; *n* = 38 cells from 10 independent experiments), and whilst the average apparent brightness was not significantly different to basal, there was a subset (4/39) of cells that displayed an increased apparent brightness (1.69–1.81). The region selected for analysis in these cells may represent an area with a high population of clusters or higher-order oligomers of CXCR4, which, whilst still not bright enough to be visible by confocal microscopy, can be resolved via their brightness in N&B. Apparent brightness decreased following pre-incubation with IT1t (1.26 ± 0.01; coefficient of variation 5.10 *n* = 38 cells from 10 independent experiments), which was significantly different to that seen with the CXCL12 treatment. Neither CXCL12 nor IT1t influenced average apparent number with respect to vehicle-treated cells, nor was there any correlation of apparent number with apparent brightness.

### 3.6. Fluorescence Recovery After Photobleaching (FRAP)

**Figure 9:**
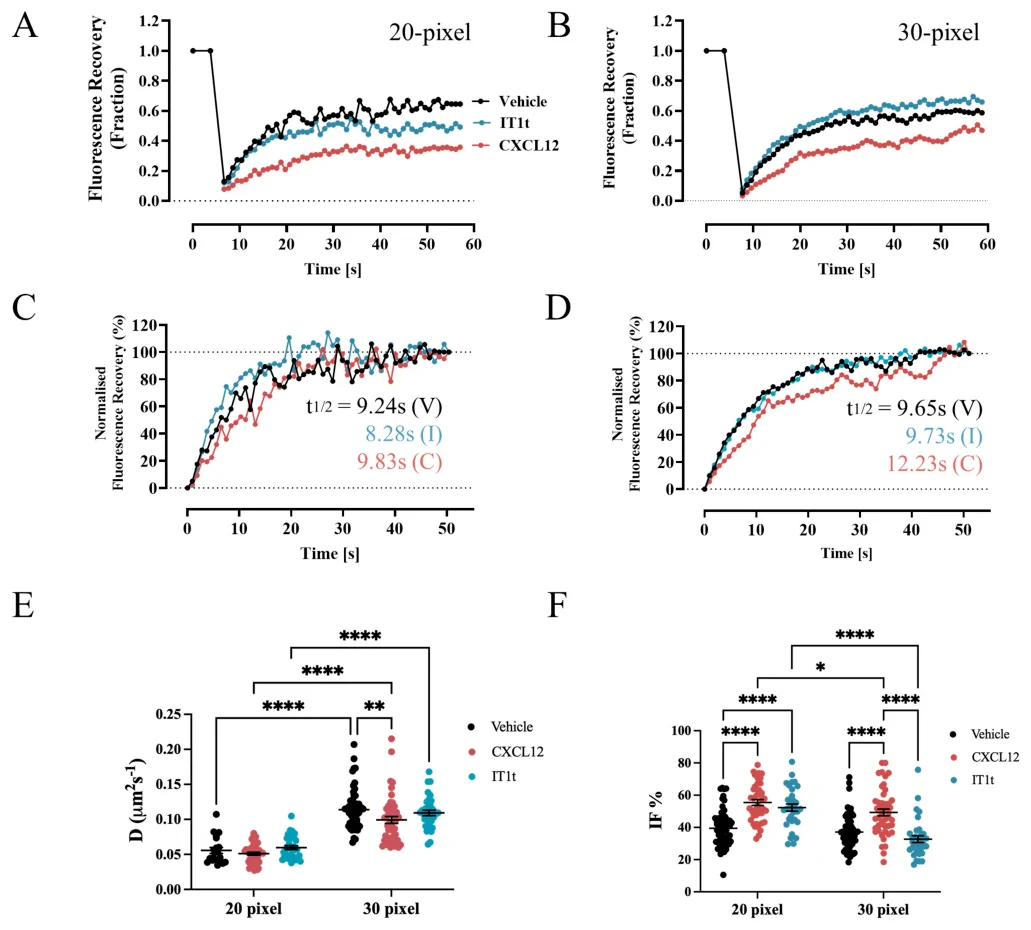
Diffusion coefficient and immobile fraction as derived from FRAP. Representative fluorescence recovery curves for (**A**,**C**) 20- and (**B**,**D**) 30-pixel bleaching regions as (**A**,**B**) a fraction of fluorescence before bleaching where time is from the start of the experiment or (**C**,**D**) normalised to the final fluorescence recovered, where the time is following bleach. The curves were constructed from 3 sample cells treated with vehicle (V, black), 10 nM CXCL12 (C, red) or 1 µM IT1t (I, blue). (**E**) Diffusion coefficient, D, and (**F**) immobile fraction, IF, derived for vehicle- (*n* =57–59), CXCL12- (*n* =44–48), and IT1t- (*n* =32–35) treated cells subject to a bleaching area of 20- or 30-pixel diameter, respectively. Two-way ANOVA followed by Šídák’s multiple comparisons test; (**E**) bleach area—*p* < 0.001 (F = 234.0, DFn = 1, DFd = 230); treatment—*p* < 0.02 (F = 3.91 DFn = 2, DFd = 230); (**F**) bleach area—*p* < 0.0001 (F = 38.2, DFn = 1, DFd = 268); treatment—*p* < 0.0001 (F = 34.6 DFn = 2, DFd = 268). (* *p* < 0.05, ** *p* < 0.01, ****p < 0.0001), mean data ±SEM are displayed.

To analyse very slow-moving or stationary SNAP-CXCR4, we used FRAP, as these remain “invisible” to FFS based analysis, which requires movement and diffusion of the fluorescent species of interest to create the fluctuations in intensity required for analysis. FRAP measurements using circular bleach areas with diameters of 20 and 30 pixels (areas: 20 pixel = 5.89 µm^2^ and 30 pixel = 13.26 µm^2^) were performed on the basal membrane of labelled HEK293G SNAP-CXCR4 cells treated with vehicle, CXCL12 (10 nM, 10 min, 37 °C) or IT1t (1 μM, 30 min, 37 °C). Fluorescence recovery curves of the bleached area fit well to a single-component 2D model of diffusion (Figure 9A), allowing the determination of the t_1/2_ value and derivation of the diffusion coefficient. The data indicate that approximately 37–39% of SNAP-CXCR4 was immobile over the scale of the bleach area. Whilst the diffusion coefficient of SNAP-CXCR4 under basal conditions increased with bleach area (Table 4, Figure 9E, 20 px; 0.053 ± 0.002 µm^2^s^−1^, 30 px; 0.11 ± 0.004 µm^2^s^−1^ *n* = 57–59 from six individual experiments), the immobile fraction remained consistent (Figure 9F, 20 px; 39.5 ± 1.5%, 30 px; 37.1 ± 1.5%). Following stimulation with CXCL12, the immobile fraction in both bleach areas increased (Figure 9F, 20 px; 55.4 ± 1.8%, 30 px; 49.3 ± 2.1% *p* < 0.0001) compared to the vehicle, indicating an agonist-mediated slowing and movement to immobile locations in the membrane. The diffusion coefficient of SNAP-CXCR4 was also slightly but significantly slowed compared to the vehicle, but this was only apparent in the larger bleached area (Figure 9E, 20 px; 0.051 ± 0.002 µm^2^s^−1^, 30 px; 0.099 ± 0.005 µm^2^s^−1^ *p* < 0.01, *n* = 44–48 from a minimum of five independent experiments). Pre-incubation with the inverse agonist IT1t did not affect the diffusion coefficient at either bleach size, but interestingly the immobile fraction was significantly increased in the smaller bleach area compared to the vehicle (Figure 9F, 20 px; 52.3 ± 2.3% *p* < 0.0001, *n* = 32 from five independent experiments). In the larger bleach area, the immobile fraction was also significantly decreased compared to CXCL12 treatment (Figure 9F, 30 px; 31.8 ± 2.1% *p* < 0.001, *n* = 35 from five independent experiments).

## 4. Discussion

Understanding the spatial dynamics of GPCRs within cell membranes is crucial for a full understanding of their signalling, trafficking and regulation. In this study, we have determined the membrane dynamics of a SNAP-tag-labelled GPCR, SNAP-CXCR4, after treatment with an agonist, CXCL12, and an inverse agonist, IT1t, by applying complementary FFS techniques in conjunction with FRAP. Along with single cell studies, which allow the exploration of heterogeneity in cell populations, FFS techniques can determine the effects of ligand stimulation not visible through standard imaging approaches. Whilst these standard approaches can deliver an essential cell-wide overview of receptor location [46,53], the fine spatiotemporal resolution is masked. We show here that we can observe ligand-mediated changes in SNAP-CXCR4 diffusion speed, particle number, and oligimerisation/clustering that occur prior to significant receptor internalisation and are dependent on the scale of the observation area. Since such FFS techniques are increasingly accessible using standard confocal microscope hardware, we show that FFS/FRAP provides the sensitivity to build a detailed picture of CXCR4 plasma membrane organisation prior to receptor internalisation.

### 4.1. Comparison of SNAP-CXCR4 Diffusion Across Scales

FCS, RICS and FRAP successfully quantified SNAP-CXCR4 diffusion (D/µm^2^s^−1^) in the plasma membrane of a model cell line. The FCS-derived diffusion coefficient, D_FCS_, for SNAP-CXCR4 under basal conditions (0.29 ±0.01 µm^2^s^−1^), was in line with that previously reported for other SNAP-labelled Class A GPCRs expressed in HEK293 cell lines [46,52]. Of the modalities employed, FCS provides the most sensitive measure of diffusion coefficient [33,54]. The high signal to noise afforded by FCS provides the statistical power to model a trace with multiple components [30], therefore separating signal from the species of interest, provided non-target species have sufficiently different dwell times. As well as the membrane-restricted SNAP-CXCR4 (τ_Di_ of ~20–70 ms), FCS can delineate signals arising from the fluorophore triplet state (τ_Di_ limited to <10 µs) and free SNAP AlexaFluor™488 dye (τ_Di_ between 20–80 µs). Furthermore, the diffraction-limited observation volume, ~0.25 fL, allows the quantification of species from the femto- to high nano-molar range, with a higher sensitivity at lower concentrations [28,29]. Like FCS, RICS also records fluorescence fluctuations arising from single molecules. The raster scanning nature of the process and the laser dwell time of 8.24 µs/pixel reduces the sensitivity to resolve multiple species, but instead provides information from a larger area and may detect slower species given the reduced photobleaching [33,55]. The diffusion coefficient derived by RICS, for SNAP-CXCR4 under basal conditions (D_RICS_; 0.20 ± 0.02 µm^2^s^−1^), was significantly slower than that from FCS (D_FCS_; 0.29 ± 0.01 µm^2^s^−1^; unpaired *t*-test, *p* < 0.0001), alluding to the presence of a slower diffusing species not visible to standard FCS. It should be noted, however, that D_FCS_ was determined from the apical membrane and D_RICS_ from the basal membrane, and we cannot rule out that the difference may be due to the way in which CXCR4 is organised at adherent and non-adherent surfaces. Whilst FCS measurements at the basal membrane are possible, high levels of scattering from the coverslip can worsen the signal-to-noise ratio and cause aberrations in the shape of the observation volume.

In contrast to FCS and RICS, FRAP determines diffusion coefficients over a larger area, and involves the physical bleaching of the fluorescent molecule of interest. FRAP diffusion speeds can therefore be underestimated due to the corona effect; the diffusion of bleached species in/out of the ROI during bleaching causes a corona around the bleach area, increasing recovery time [56,57]. Whilst additional corrections could be applied to derive the absolute D [56,57], FRAP can be a straightforward method to investigate comparative effects on species diffusion following treatment. The D_FRAP_ values for SNAP-CXCR4 were 0.05–0.11 µm^2^s^−1^, depending on bleach area, and measurements were sensitive to the slowing of D_FRAP_ following CXCL12 treatment (Figure 9E). These D_FRAP_ values were consistently lower than those obtained with D_FCS_ and D_RICS_. Derivations of D from FRAP will differ depending on the size and shape of the bleach region unless completely free diffusion is observed [58,59]. This was illustrated in our experiments, where the diffusion of fluorescent species under vehicle conditions was measured as faster in the larger (Figure 9E, 30 pixel) bleach area as compared to the smaller (20 pixel). This increase in SNAP-CXCR4 D_FRAP_ with bleach area illustrates that both lateral diffusion and cytosol-membrane exchange play a part in the regulation of CXCR4 in the plasma membrane [60].

Several factors underlie the differences in D observed between techniques. Within the live cell environment, D_RICS_ and D_FRAP_ will not deliver the sensitivity to resolve the diffusion coefficient of multiple species on the plasma membrane, in part due to the delicate balance of employing a detection laser power that delivers sufficient signal to noise with limited bleaching [61]. The spatial nature of FRAP and RICS means that the derived D_FRAP_ and D_RICS_ will incorporate multiple modes of molecular movement—including lateral diffusion, hindered or restricted diffusion, active receptor internalisation, and the passive exchange of fluorescent species between the membrane and cytosol. The relative contribution of each mode, and hence the resulting D, is dependent on the spatial and temporal scale of observation and on any external perturbations (e.g., ligand stimulation). Because FRAP relies on an ensemble fluorescence recovery measurement of D and operates with a micron-level bleach spot as opposed to a diffraction-limited confocal volume, D_FRAP_ cannot directly be compared like-for-like with D_FCS_; indeed, these techniques often yield quite different results due to underlying methodological and physical differences [62,63]. Several structural elements within the complex live cell environment—including lipid rafts, cytoskeletal architecture, immobile domains, membrane curvature and phase domains—can cause apparent D_FCS_ and D_FRAP_ to appear faster or slower, respectively [63,64]. FCS measurements are further biased towards faster-moving species, as slower species are more likely to be bleached during acquisition, and may not be adequately represented in the trace [55].

### 4.2. Ligand-Regulated SNAP-CXCR4 Movement Implies an Underlying Spatial Organisation

Unlike FFS techniques, FRAP can detect very slow or stationary species, termed the immobile fraction. Since FCS and RICS (and their associated techniques, PCH and N&B) rely on the analysis of fluctuations in detected intensity, anything stationary will simply add to background fluorescence or will be bleached. These slow and stationary elements are routinely removed to allow accurate data fitting in FFS analysis, either through cropping the initial decrease in mean intensity (FCS traces) or via a rolling frame average (RICS/N&B).

In our study, treatment with agonist caused an increase in the SNAP-CXCR4 immobile fraction in both bleach areas, indicating a significant movement of agonist-stimulated CXCR4 to immobile regions of the membrane. This was accompanied by a small but significant agonist-induced decrease in D_FRAP_ (in the larger bleach area). Together, these results suggest an underlying change in the spatial organisation of SNAP-CXCR4, which only becomes apparent at larger observation scales [59,60]. Such spatial organisation could include the presence of structural features, e.g., caveolae [63] and lipid rafts [40], multi-protein clusters [65], or indeed interactions with the cytoskeleton [66]. The ligand-mediated spatial reorganisation of other class A GPCRs has previously been revealed through the application of FRAP [52,67]. The immobile fraction of the µ-opioid receptor was shown to increase following agonist treatment, which was attributed to the interaction of ligand-stimulated receptors with other proteins and co-receptors, and allowed pre-internalisation mechanisms to be isolated [52]. Similarly, the Neurokinin 2 receptor was demonstrated to exist in both free and confined diffusional states [67], with the latter theorised to be the receptor transiently associating with scaffolding proteins before being localised to clathrin-coated pits following ligand stimulation. Through image-based methodologies, single particle tracking (SPT) and single molecule microscopy, ligand-driven effects on CXCR4 mobility have already been linked to clathrin-dependent endocytic structures and the actin cytoskeleton [21,68], incorporating both diffusivity and stoichiometry inferences.

### 4.3. CXCL12 Drives CXCR4 Clustering

The stoichiometric organisation of receptors into monomers, dimers and oligomers can play a crucial role in regulating receptor function and trafficking [23,24,25]. The clustering of CXCR4 has been shown to affect receptor activity [20] and to have functional effects regarding cell migration [21] and cell survival [69]. This organisation has also been shown to be ligand-driven by agonists and/or antagonists alike [20,21,70]. Within our study, PCH and N&B analyses provide information on the brightness of the detected species, which can inform on receptor stoichiometry within the system. Again, these readouts describe different scales of observation, PCH provides information on the nanoscale (~0.25 fL), whereas N&B can provide a microscale map of average apparent brightness (B), representative of the average stoichiometry of the species population. A population in which species are forming higher-order oligomers, clusters, or indeed an increase in the number of clusters, will result in an increase in the average brightness per ‘particle’ detected. Due to its sensitivity, PCH can resolve multiple populations that differ in brightness, as observed with the ‘dimmer’ and ‘brighter’ components of the SNAP-CXCR4 traces (Figure 6). Since monomers and dimers cannot usually be resolved by differences in diffusion (a doubling in diffusion time requires an 8-fold increase in object radius; Equation (1)), combining FCS and PCH can be extremely powerful. Following CXCL12 treatment, SNAP-CXCR4 slows (D_FCS_, D_FRAP_) and increases in both brightness and heterogeneity, consistent with a pre-clustering of receptors, which may precede internalisation. This may also precede a further slowing (e.g., to clathrin pits), which is detected as IF in FRAP, but not visible in FCS/PCH. Martínez-Muñoz (2018) [21] et al. also observed a reduction in diffusivity and an increase in CXCR4 clusters following CXCL12 treatment in JKCD4 cells via SPT. Their data show that following CXCL12 treatment, these clusters formed a larger component of the immobile tracked species.

### 4.4. Limitations and Biological Interpretation

FFS techniques are extremely sensitive and can deliver powerful data with high spatiotemporal resolution. However, when applied to complex systems such as live cells, biological interpretation is complex. Precise stoichiometric determinations of receptor oligomers and clusters can be challenging, especially when dynamic and transient interactions (e.g., GPCR oligomerisation [71]) are being studied. In this study, measured PCH and N&B brightness values will not precisely correspond to integer orders of oligomer magnitude, but rather the average stoichiometry within a dynamic and heterogeneous species population. Biological interpretation will rely on examining a shift towards increased or decreased brightness as an indication of a shift to an increased or decreased oligomeric species. Within cell-based systems, molecular brightness can also be underestimated, due to factors such as environmental quenching from other membrane proteins, pH changes (e.g., in endosomes), or proximity to other fluorophores (e.g., clustered SNAP-CXCR4). Conversely, commercial software tends to use a 3D PCH model, which may overestimate the molecular brightness of a 2D constrained species such as SNAP-CXCR4. Oligomeric controls of species known to be obligate monomeric or set higher order (e.g., dimeric) species can help to delineate the population [20,70]. However, other techniques such as stepwise bleaching [72,73] may be required for absolute quantification.

By using a fluorescence system, we rely on efficient labelling, and despite optimisation, no system confers 100% labelling, including SNAP-tag [74]. The quantification of particle number and oligomer size through brightness may therefore be underestimated, but, with the assumption that labelling efficiency is independent of oligomeric state, relative proportions of different components should remain the same, and ligand-induced effects are relative to that recorded from vehicle conditions. Genetic modification to introduce a protein tag necessitates functional characterization, and as well as effects on localisation and function (see Section 3.1), the size of the labelling system should be considered. Empirically, D_FCS_ could resolve a minimum 4-fold difference in molecular weight [30]; therefore, the SNAP-tag itself (19.4 kDa) should have a negligible effect on native CXCR4 (65–80 kDa) diffusion. We previously demonstrated no difference in D_FCS_ for the native Adenosine-A_3_ receptor and a GFP-tagged variant [75]. The peptide CXCL12 (8 kDa) and the small molecule IT1t (0.48 kDa) are also sufficiently small to have a negligible effect on species size through binding alone, but may consequently affect diffusion via shifts in oligomerisation or by inducing trafficking [20,21,70]. Hence, the small ligand-induced changes in D are indicative of the reorganisation of CXCR4 in the membrane, whether through oligomerisation, clustering or interactions with cell scaffolding.

FCS is routinely performed on apical membranes since optical limitations near the coverslip—light scattering, worsened signal-to-noise ratio and observation volume aberrations—make basal membrane reads more difficult. Similarly, RICS/FRAP require the flatness of the basal membrane. By necessity, this means FCS and FRAP data are not obtained from the same cell area. For an HEK293 cell, which is non-polarised, this may not be of significant consequence, but in polarised cells such as epithelial-derived models, this may be a significant consideration.

Despite these limitations, the high spatial resolution delivered by FFS at a single cell level provides insight into subtle biological events, which are invisible at the confocal level and when examining cell populations. By unpicking the inferences of each technique, new hypotheses can be generated and tested with more specialised hardware and techniques. Dependent on the focus, these techniques may include SPT [21] and Stimulated Emission Depletion (STED)-FCS to identify modes of diffusion such as free, hop and hindered diffusion [76] or stepwise bleaching [72,73] to interrogate stoichiometry.

## 5. Conclusions

In conclusion, we have employed FFS techniques (FCS/PCH/RICS/N&B), alongside FRAP, to explore the ligand-driven reorganisation of SNAP-CXCR4 at early time points that are not detectable with standard confocal imaging. We show that the stimulation of CXCR4 with the endogenous agonist CXCL12 induces receptor clustering and a slowing of diffusion, including an increase in the amount of immobile receptor. These suggest a significant change in the organisation of CXCR4 in the membrane, which occurs prior to the CXCL12-stimulated internalisation of CXCR4. Overall, this study emphasises the feasibility of using FFS approaches in concert across different spatial scales to study the membrane organisation of proteins not revealed by standard imaging.

## Figures and Tables

**Table 1:** Diffusion coefficient (D), particle number (N) and molecular brightness (ε) as derived from FCS and PCH analysis. % Fit refers to the number of traces for which a 1-component fit was preferred. Data are mean ± S.E.M from *n* cells over 6–10 independent experiments. D_FCS_ is significantly different between CXCL12 and IT1t treatment; one-way ANOVA followed by Tukey’s multiple comparisons test * *p* < 0.05.

	FCS			PCH		
Treatment	D_FCS_ (μm^2^s^−1^)	N (μm^−2^)	*n*	ε (Dimmer Component, kHz Count/Molecule·s^−1^)	% Fit with 1-Component	*n*
Vehicle	0.29 ± 0.011	230 ± 10	37	41.34 ± 3.47	97	31
10 nM CXCL12	0.27 ± 0.011 *	253 ± 15	31	33.14 ± 3.13	66	31
1 µM IT1t	0.32 ± 0.015 *	260 ± 18	31	39.26 ± 3.53	94	31

**Table 2:** Diffusion coefficient and species amplitude as derived from RICS. Data are mean ± S.E.M. Species amplitude is significantly different between CXCL12 and IT1t treatment, one-way ANOVA ** *p* < 0.01.

Treatment	D_RICS_ (μm^2^s^−1^)	Species Amplitude	*n*
Vehicle	0.20 ± 0.02	73.9 ± 12.5	18
10 nM CXCL12	0.22 ± 0.01	55.2 ± 6.3 **	28
1 µM IT1t	0.20 ± 0.01	91.4 ± 7.1 **	31

**Table 3:** Apparent number and apparent brightness as derived from N&B analysis. Data are mean ± S.E.M. Apparent brightness is significantly different between CXCL12 and IT1t treatment, as tested with one-way ANOVA followed by Tukey’s multiple comparisons test ** *p* < 0.01.

Treatment	Apparent Brightness	Apparent Number	*n*
Vehicle	1.31 ± 0.02	10.0 ± 0.72	18
10 nM CXCL12	1.34 ± 0.03 **	8.41 ± 0.51	39
1 µM IT1t	1.26 ± 0.01 **	9.15 ± 0.75	38

**Table 4:** t_1/2_, diffusion coefficient and immobile fraction derived from FRAP. Data are mean ± S.E.M. Results of statistical analysis by 2-way ANOVA are displayed on Figure 9.

Bleach Area	20 Pixel				30 Pixel			
Treatment	t_1/2_ (s)	D_FRAP_ (μm^2^s^−1^)	IF (%)	*n*	t_1/2_ (s)	D_FRAP_ (μm^2^s^−1^)	IF (%)	*n*
Vehicle	9.47 ± 0.34	0.053 ± 0.002	39.5 ± 1.5	59	9.76 ± 0.30	0.11 ± 0.004	37.1 ± 1.5	57
10 nM CXCL12	9.86 ± 0.44	0.051 ± 0.002	55.4 ± 1.8	44	11.64 ± 0.49	0.099 ± 0.005	49.3 ± 2.1	48
1 µM IT1t	8.33 ± 0.36	0.060 ± 0.002	52.3 ± 2.3	32	10.06 ± 0.37	0.11 ± 0.004	31.8 ± 2.1	35

## Data Availability

The original contributions presented in this study are included in the article/Supplementary Materials. Further inquiries can be directed to the corresponding authors.

## Supplementary Materials

The following supporting information can be downloaded at: https://www.mdpi.com/article/10.3390/biom16081107/s1, Figure S1: (A) Representative autocorrelation curve and diffusion model fit to compare 1- and 2-component fit. Raw data (black line)and Autocorrelation fit (red line)with and without a second component. Deviation from fit is displayed below autocorrelation curve. (B) Percentage of free SNAP dye (SNAP-AF488) and SNAP-tagged receptor(SNAP-CXCR4-AF488) following 2-component fit. Each data point represents a single cell, n = 25 cells subject to vehicle treatment; Table S1: % of species that comprise the second component when a 2-component model is preferred. Data are mean ± S.E.M where applicable with n = number of single cell measurements.
