# Supplementary material for: Characterising C-X-C Chemokine Receptor 4 Dynamics in the Cell Membrane Using Fluorescence Fluctuation Spectroscopy
Source: Biomolecules. 2026 Jul 29;16(8):1107. doi: 10.3390/biom16081107 (PMC13511381; doi:10.3390/biom16081107)
Supplement: Supplementary file 1 [file biomolecules-16-01107-s001.zip › biomolecules-4424328-supplementary.pdf]

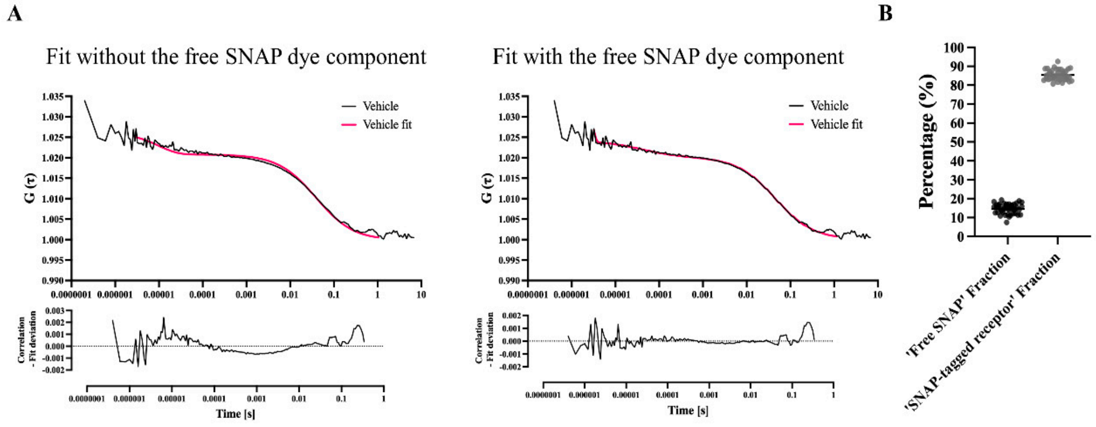

Supplementary Figure S1. (A) Representative autocorrelation curve and diffusion model fit to compare 1- and 2-component fit. Raw data (black line)and Autocorrelation fit (red line)with and without a second component. Deviation from fit is displayed below autocorrelation curve. (B) Percentage of free SNAP dye (SNAP-AF488) and SNAP-tagged receptor(SNAP-CXCR4-AF488) following 2-component fit. Each data point represents a single cell, n=25 cells subject to vehicle treatment.

Supplementary Table S1. % of species that comprise the second component when a 2-component model is preferred. Data are mean ± S.E.M where applicable with n = number of single cell measurements.

Component 2	Vehicle	IT1t	CXCL12
%	4.09	3.12 ± 0.12	15.67 ± 7.29
n	1	2	11
